# Cancer complaints: The profile of patients from the emergency department of a Brazilian oncology teaching hospital

**DOI:** 10.12688/f1000research.12632.1

**Published:** 2017-10-31

**Authors:** Felipe Batalini, Millena Gomes, Fábio I, Flávio Kuwae, Giselle Macanhan, Julio L.B. Pereira

**Affiliations:** 1 Department of Medicine, Boston University Medical Center, Boston, MA, USA; 2 Hospital Araújo Jorge, Goiânia, GO, Brazil; 3 Department of Medicine, Universidade Federal de Goiás, Goiânia, GO, Brazil; 4 Department of Surgery, Hospital Beneficência Portuguesa de São Paulo, São Paulo, SP, Brazil

**Keywords:** cancer, complaints, neoplasms, pain, oncology, hospital

## Abstract

**Background:** With the increase in prevalence of cancer in our society, we aim to clarify through primary data use what drives emergency department (ED) utilization among patients with cancer.

**Methods:** This is a cross-sectional study. A direct survey was applied to cancer patients over 277 visits in 2015. Variables including chief complaint for current and last visit, frequency of visits, primary tumor site, and demographics were collected.

**Results:** Pain was the most common complaint, responsible for 40% of visits, followed by constitutional symptoms (17%), and gastrointestinal complaints (11%). Abdominal pain was the single most noted pain type, with 18.4%, and had the highest rate of recurrence. It was followed by back pain, dyspnea, asthenia and fever, accounting for 8.5%, 8.5%, 8.1% and 7%, respectively. Cervical cancer represented 14.8% of patients, followed by breast (11.6%) and lung (7.6%) cancers. The majority of patients visited the emergency department less than once a month.

**Conclusion: **The drivers of emergency department utilization among patients with cancer found through primary use data mostly confirm findings from larger studies with secondary use data. Our research underscores the burden of pain to patients with cancer, as it is the most common complaint leading to ED visits, and generally requires multiple visits. Abdominal pain was more likely to recur than other complaints. Patients could benefit from focused outpatient pain management, and from more research and education targeting cancer-related pain.

## Introduction

The progressive increase in life expectancy of cancer patients, which is associated with the development and availability of newer more effective therapies has raised the prevalence of cancer in our society ^1, 2^. Even though recent therapies, such as immunotherapy, tend to have an improved side-effect profile ^3^, patients still suffer from stigma and progression of the disease, especially those with incurable conditions. Good outpatient care is a crucial component of the treatment of the oncologic patient, and emergency department (ED) visits are a strong indicator of low quality of life among cancer patients ^4^. Other authors have studied the profile of cancer patients in general hospitals and through registries ^5– 9^. The use of secondary data can sometimes lead to information bias ^10^. We aim to understand what drives ED utilization among cancer patients in an oncology teaching hospital using primary source data.

## Methods

### Study participants

This is a cross-sectional study. We analyzed survey data from 277 patients from Araújo Jorge Hospital, a major oncology-only teaching hospital located in the city of Goiânia, Goiás, Brazil. After approval by the institutional review board (IRB approval number: CAAE: 43909215.7.0000.0031), written informed consent was obtained from patients, or from caregivers for very debilitated patients, for participation in the study. The only inclusion criterion was arrival at the ED, and the only exclusion criterion was the refusal to participate in the study. Data was collected upon arrival at the ED for 12 consecutive days, 24 hours a day, in May 2015. The questionnaire is provided as Supplementary File 1. Medical records were used to obtain specific demographical information only when necessary. There was potential of recall bias for information regarding prior visits. In order to minimize information bias, all the authors reviewed all the data and whenever there was doubt in categorization of chief complaints, consensus was achieved before final categorization.

### Data variables and analysis

It is important to remark that some patients presented at the ED multiple times; therefore, the variable unit is the patient visit and not the patient itself, so frequencies and proportions will reflect those. The primary study variable is the chief complaint, but we also collected other variables such as gender, age, main complaint, primary tumor site, city of origin, age at diagnosis, insurance type, frequency of visit to the ED, time and reason for the previous visit.

Descriptive analysis of data was performed through SPSS version 24, from IBM.

## Results

Patient demographics are shown in Table 1.

**Table 1.** Patient demographics of oncologic patients from a Brazilian oncology teaching hospital when presenting at the emergency department.

Demographic	Value
Age, years (SD, range)	59 (14, 10–100)
Age at diagnosis, years (SD)	57 (14)
Male gender (%)	48
Type of insurance (%) - Sistema Único de Saúde - Private	95.2 4.8
Place of origin (%) - Goiânia (city) - Goiás (state) - Brazil (country)	50.2 92.1 99.3

### Chief complaints

**Figure 1.**
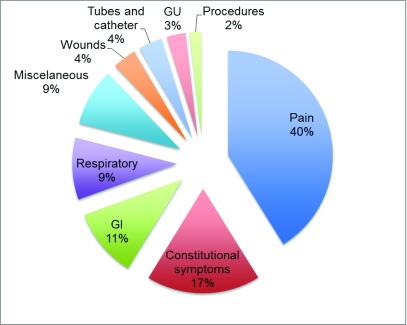
The most common chief complaints at arrival in the emergency department of an oncologic hospital. GI, gastrointestinal; GU, genitourinary.

Pain was the most common complaint in their presentation to the emergency department, accounting for 40.4% (n=112) of all visits ( Figure 1). Constitutional symptoms were second with 17.3% (n=48) of visits. Gastrointestinal-related complaints were third with 10.5% (n=29). Respiratory symptoms were the fourth most common complaints with 9.4% (n=26) of all visits. Altogether, these four most common complaints formed 77.6% (n=215) of all visits. Less common were complaints related to wounds (3.6%, n=10), malfunctioning of tubes and catheters (3.6%, n=10), genitourinary complaints (2.9%, n=8), visit for procedures (1.8%, n=5) and others not specified (8.7%, n=24).

Among all pain-related complaints, abdominal pain was the most common corresponding to 44.6% of those cases, followed by back pain with 20.5%. Constitutional symptoms accounted for 17.3% of all visits; divided into asthenia, fever, anorexia and malaise, responsible respectively for 46%, 40% and 12.5% of the complaints in this category. From the gastrointestinal complaints, nausea and vomit were responsible for 48% of cases, followed by diarrhea (17%) and constipation (14%). Within the respiratory category, 88% of complaints were dyspnea, more common than cough and hemoptysis.

In individual complaint analysis ( Table 2), as opposed to analysis by categories ( Figure 1), the most common finding was abdominal pain with 18.4% (n=50); following was back pain (8.5%, n=23), dyspnea (8.5%, n=23), asthenia (8.1%, n=22), fever with (7.0%, n=19) and vomiting and nausea (4.8%, n=13).

**Table 2.** Most common chief complaints of oncologic patients from a Brazilian oncology teaching hospital when presenting at the emergency department.

Chief complaint	n	%
Total	272 ^*^	100
Abdominal pain	50	18.4
Back pain	23	8.5
Dyspnea	23	8.5
Asthenia	22	8.1
Fever	19	7.0
Nausea and vomit	13	4.8
Wounds	10	3.7
Tubes and catheters	10	3.7
Anorexia	6	2.2
Diarrhea	5	1.8
Procedures	5	1.8
Constipation	4	1.5
Vaginal bleeding	4	1.5
Urinary symptoms	4	1.5
Hematochezia	3	1.1
Others, less common	71	26.1

*Missing data on chief complaint in 5 of 277 interviewed patients.

For patients who reported a previous ED visit, the chief complaint at the last visit was recorded and from the 192 visits amenable for analysis, 55.2% (n=106) patients were returning to the ED with the same complaint as the last visit. From those presenting with pain, 79.7% (59/74) had a prior visit for the same reason. Those presenting with abdominal pain described abdominal pain as the chief complaint at their last visit in 85.7% (24/28) of times.

### Primary neoplasia

Table 3 shows the most common neoplasias. Cervical cancer was the most frequent primary tumor, accounting for 14.8% (n=41) of all visits during the study period. Breast cancer was second (11.6%, n=32), followed by lung (7.6%, n=21) and colorectal cancers (7.6%, n=21). Prostate and esophageal cancer formed 5.4% (n=15) each. Gastric cancer: 3.2% (n=9), followed by liver and pancreatic cancers, each one of them with 1.4% (n=4) of cases.

**Table 3.** Most common neoplasias presenting at the emergency department of a Brazilian oncology teaching hospital, categorized by organs.

Primary neoplasia by organs	%
Uterine/cervical	14.8
Breast	11.6
Lung	7.6
Colorectal	7.6
Prostate	5.4
Esophageal	5.4
Gastric	3.2
Liver	1.4
Pancreas	1.4
Other	41.6

In analysis by biological systems ( Table 4), genitourinary tract cancers were the most common primary neoplasms (26.3%, n=73), driven mostly by cervical cancer with 56.1% (n=41) within this category. Gastrointestinal neoplasias constituted 20.6% (n=57) of all visits. Head and neck cancers were 15.5% (n=43), where laryngeal cancer corresponded to 3.2% (n=9), followed by tongue cancer with 1.8% (n=5). Hematologic malignancies accounted for 5.8% (n=16) cases, within these: 4% (n=11) were lymphomas. Primary skin cancers accounted for 2.9% (n=8) of the visits, among which the most common tumor was melanoma, representing 2.2% (n=6) of total cases. Central nervous system cancers constituted 2.2% (n=6), from which only 1.4% (n=4) were primary.

**Table 4.** Most common neoplasias presenting at the emergency department of a Brazilian oncology teaching hospital, categorized by biological systems.

Primary neoplasia by systems		%
1	Genitourinary tract	26.3
2	Gastrointestinal	20.6
3	Head and neck	15.5
4	Hematologic	5.8
5	Breast	11.6
6	Lung	7.6
7	Skin	2.9
8	CNS	2.2
9	Other	7.5

### Time of last visit and frequency of visits

According to patients, 19.3% (n=52) had visited the ED for the first time, and 13.0% (n=35) had come before in the prior month. 10.0% (n=27) stated that their last visit was the day before. This matches the finding of 8.2% (n=22) reporting visiting the ED daily. Overall, the most reported frequency was “less than once a month” (50.2%, n=139).

### Time of arrival

The 277 patients were seen over 288 consecutive hours, average 0.96 patients per hour. Most of them (71.1%, n=197) arrived during day shifts, defined from 7 am to 6:59 pm, and the minority (28.9%, n=80) came at night shifts, from 7 pm to 6:59 am. The busiest time was between 10 am and 10 pm, with average of 1.52 patient per hour, when 79.1% (n=219) arrived. In contrast, the period from 10 pm to 10 am had an average of 0.48 patients per hour.

Cancer patients presenting at the emergency department Click here for additional data file. Copyright: © 2017 Batalini F et al. 2017 Data associated with the article are available under the terms of the Creative Commons Zero "No rights reserved" data waiver (CC0 1.0 Public domain dedication).

## Discussion

This study registered 277 consecutive ED visits to Araújo Jorge Hospital, an oncology-only teaching hospital. The main limitations of the study are the absence of staging information and the fact it is based in a single institution. Its strength resides in the quality of data - collected for primary use - and despite the limited number of visits, it shows that pain is the main driver of patients with cancer to the ED, corroborating findings of previous studies with larger numbers from secondary use data ^6– 9^. Demographic analysis showed that the hospital is a strong regional reference for oncologic care, serving patients not only from the mid-west but also from the north and northeastern regions. There were no patients from the south or southeastern regions of the country.

Abdominal pain was the most common single complaint with 18.4% of all visits; patients with these complaints were more likely to return with the same complaint than others, leading to more frequent visits than those with different complaints. One possible explanation is that abdominal pain unites complications from many different organs and systems, including tumors from the most common sites (see Table 4). Also, its higher frequency can be at least in part explained by the higher frequency of cervical cancer in our population, which commonly complicates with intra-abdominal and pelvic metastasis.

Complaints at the current visit was the same as the last visit in 55.2% of times, revealing the opportunity to predict the chief complaint of future visits, especially in the case of abdominal pain. In practice, nausea and vomit are major complaints of this population, but it ranked only sixth in this study, this is possibly explained by either easier good outpatient control or not enough severity to bring patients to the hospital.

In our study, cervical cancer had the highest frequency in the ED, with 14.8% (n=41) of all cases, followed by breast, lung and colorectal cancers, with 11.6% (n=32), 7.6% (n=21) and 7.6% (n=21), respectively. Another Brazilian study, Borges *et al.* ^6^ also found cervical cancer as the most common at the ED, and almost two-thirds of the patients had one of the following primary tumors: urological, breast, gastrointestinal tract and lung cancer. In contrast, most studies found lung cancer as the major driver of visits, followed by breast and colorectal tumors ^5, 7, 11, 12^. This inequality is associated with low-resource settings with suboptimal programs such as screening and vaccination ^13^. According to The Brazilian Cancer National Institute (INCA), cervical cancer has high incidence and prevalence in Brazil, and it is estimated to be responsible for 70% of the total of uterine cancer. Furthermore, although it’s ranked third in incidence in the country, it is the second in the mid-west region of the country, where this study was performed, responsible for 11.4% of all female malignancies. In parallel, in the US, only 17% of uterine cancers are expected to originate from the cervix, according to public domain reports from the National Cancer Institute Surveillance, Epidemiology, and End Results Program. This disparity should encourage more focus on preventive measures, such as better vaccination rates against HPV and Pap smear coverage in Brazil ^13^. Despite not being the most incident, melanoma was the most common skin cancer leading to ED usage, likely due to its higher aggressiveness and invasion potential than other skin cancers.

We found a high rate of patients with multiple visits, including 10% of patients reporting daily visits. More than half of patients (63.8%, n=172) visited the ED in the previous month. These are findings that differ from Leak *et al.* ^12^, who showed that 71% of the patients had visited the ED only once before their death. The absolute majority (95%) of patients were insured by Sistema Único de Saúde (SUS), the Brazilian public health system, therefore suggesting low-income population, with limited access to costly pain medications; requiring some of our patients to come to the ED on a daily basis for analgesia. In addition, cultural behavior limits goals of care discussions, causing a significant barrier to adequate end-of-life care. In this scenario, the expansion of the role of pain clinic and palliative care initiatives could immensely benefit patients by easing the dying process. Good outpatient symptom control could lead to decrease of ED utilization. Furthermore, this study once again highlights the importance of pain management in oncology, as a major topic in the field, and as such, it should be given extra emphasis in oncology training. More research is needed for the development of new therapies for pain in cancer patients.

## Ethical statement

The research meets all applicable standards with regard to the ethics of experimentation and research integrity, and the following is being declared true. As an expert scientist and along with co-authors of concerned field, the paper has been submitted with full responsibility, following due ethical procedure, and there is no duplicate publication, fraud, plagiarism, or concerns about animal or human experimentation.

IRB approval number: CAAE: 43909215.7.0000.0031

## Data availability

The data referenced by this article are under copyright with the following copyright statement: Copyright: © 2017 Batalini F et al.

Data associated with the article are available under the terms of the Creative Commons Zero "No rights reserved" data waiver (CC0 1.0 Public domain dedication). 

Dataset 1: Cancer patients presenting at the emergency department in Brazil. Doi, 10.5256/f1000research.12632.d182277 ^14^

## Supplementary material

Supplementary File 1: Survey in Portuguese and English.
