# Supplementary material for: Cancer complaints: The profile of patients from the emergency department of a Brazilian oncology teaching hospital
Source: F1000Res. 2017 Oct 31;6:1919. [Version 1] doi: 10.12688/f1000research.12632.1 (PMC6198257; doi:10.12688/f1000research.12632.1)
Supplement: Supplementary file 2 [file f1000research-6-13677-s0001.tgz › bef62091-60e2-4be2-8664-0d97700fb674.docx]

**Translation to English is provided in red within brackets.**

**Questionário - Queixas de Câncer: O perfil dos pacientes do Setor de Pronto-Atendimento do Hospital Araújo Jorge** [Questionnaire – Cancer complaints: The profile of patients from the emergency department of Hospital Araújo Jorge]


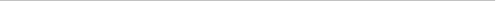


Pesquisador:[Researcher]

Data e horário:[Date and time]

Sexo: M / F [Sex: M / F]

Idade: [Age]

1. Qual o motivo da visita hoje? [What is the reason for the visit today?]

Dor: Cervical, torácica, lombar, dorsal, abdominal, epigástrica, suprapúbica, inguinal, anal, nádegas, quadris, orelhas, cabeça, membros, peito, garganta, ossos, articulações, pênis, difusa

[Pain: Neck, thorax, lumbar, back, abdominal, epigastric, suprapubic, inguinal, anal, buttocks, hip, ears, head, limbs, chest, throat, bones, joints, penis, diffuse]

Gastrointestinal: Vômitos, diarréia, icterícia, náuseas, hematêmese, ascite, constipação, disfagia, hematoquezia, sangramento retal, melena, fistula abdominal

[Gastrointestinal: Vomiting, diarrhea, jaundice, nausea, hematemesis, ascites, constipation, dysphagia, hematochezia, rectal bleeding, melena, abdominal fistula]

Respiratório: Dispnéia, tosse, dor respiratória dependente, secreção infecção pleural infecciosa, hemoptise, fístula pulmonar

[Respiratory: Dyspnea, cough, ventilator dependent pain, secretion, infection, hemoptysis, pulmonary fistula]

Lesões: Feridas de estoma, ferida operatória, deiscência de sutura, queda, trauma, fratura, corte, re-corte, erisipela, lesões herpetiformes, eritema, pápulas eritematosas, eczema

[Lesions: stoma wounds, surgical wounds, suture dehiscence, fall, trauma, fracture, cut, erysipelas, herpetic lesions, erythema, papules, eczema]

Sintomas constitucionais: Febre, astenia, caquexia, perda de peso, anorexia, inapetência, mal-estar

[Constitutional symptoms: Fever, asthenia, cachexia, weight loss, anorexia, malaise]

Genitourinário: Disúria, estrangúria, hematúria, fecaluria, sangramento vaginal, alterações de freqüência urinária, anúria, obstrução urinária, insuficiência renal, leucorréia, desconforto vulvar

[Genitourinary: Dysuria, dribbling, hematuria, fecal urination, vaginal bleeding, urinary frequency, anuria, urinary obstruction, renal insufficiency, discharge, vulvar discomfort]

Neurológico: Parestesia, paralisia, tontura, convulsões, síncope, confusão, depressão, tentativas de suicídio, diminuição do nível de consciência, estupor, abstinência alcoólica

[Neurologic: Paresthesia, paralysis, dizziness, seizures, syncope, confusion, depression, suicide attempt, altered mental state, lethargy, alcohol withdrawal]

Cardiovascular: Hipertensão, palpitações, parada cardíaca, infarto agudo do miocárdio

[Cardiovascular: Hypertension, palpitations, cardiac arrest, myocardial infarct]

Reação alérgica: Alergias, urticária, prurido, irritação nos olhos

[Allergic reaction: allergies, urticarial, pruritus, eye irritation]

Procedimentos e cuidados: Procedimentos cirúrgicos, remoção de cateter urinário, remoção de dreno de mama, retirada de pontos, substituição de cateter urinário, raio-X, troca de tubo de traqueostomia, troca de bolsa de colostomia, reavaliação de gastrostomia, transfusão, quimioterapia, fazer a gastrostomia, deiscência de sutura, secreção / sangramento ferida

[Procedures and general care: Surgical procedures, urinary catheter removal, breast drain removal, suture removal, urinary catheter exchange, radiograph, tracheostomy tube exchange, colostomy bag exchange, gastric stoma evaluation, transfusion, chemotherapy, elective gastrostomy, suture dehiscence, secretion or wound bleeding]

Outros: Anasarca, epistaxe, hiperglicemia, gengivite, linfadenopatia cervical, hemorragia, edema de membros inferiors. Inserir outro:

[Others: Anasarca, epistaxis, hyperglycemia, gingivitis, cervical lymphadenopathy, hemorrhage, lower extremities edema. Insert other:]

1. Neoplasia primária: [Primary neoplasia]
2. Cidade/ UF de origem: [City/State of origin]
3. Qual sua idade quando foi diagnosticado(a) com câncer? [How old were you when diagnosed with cancer?]
4. É atendida por algum plano de saúde? SIM / NÃO [Do you have a private health insurance? YES/ NO]
5. Com qual frequência você vem ao SPA? [How often do you come to the ED?]
6. Diariamente [Daily]
7. Semanalmente  [Weekly]
8. Mensalmente [Monthly]
9. Menos que 1x/mês [Less than once a month]
10. Quando foi a última vez que veio ao SPA? [When was the last time you came to the ED?]
11. Qual o motivo da visita na última vez que veio ao SPA? [Why did you come to the ED last time?]
