# Supplementary material for: Cancer complaints: The profile of patients from the emergency department of a Brazilian oncology teaching hospital
Source: F1000Res. 2017 Oct 31;6:1919. [Version 1] doi: 10.12688/f1000research.12632.1 (PMC6198257; doi:10.12688/f1000research.12632.1)
Supplement: Peer review history

# Peer review: Referee response for version 1

**DOI:** 10.5256/f1000research.13677.r37018

**Published:** 2018-10-19

**Authors:** Hugo Akabane

**Affiliations:** 1 UCSF Fresno, Fresno, CA, USA

The strengths of this research project relies on:

1.  The paper is well written and there is good fluidity in the text.
2.  The study was well designed and performed in a simple format, focusing in descriptive analysis, which was appropriate to its goals.
3.  It was a short study but its execution clearly required significant effort from the research team to interview patients in their arrival to the emergency department 24 hours a day and 7 days a week. This is not an ambitious project but rather important in the sense that the primary data acquired mostly validates findings from larger studies from billing codes (secondary data use).
4. The project was performed in an academic hospital from the middle west of Brazil with a huge coverage area, involving patients from many different regions of the country, and therefore represents a large patient population.
5. This project also has a clear message that patients with cancer suffer enormously from their disease and pain control should be a major topic in oncology. It underscores that patients need great support, especially at the end-of-life care.
6. The references include the main papers within the scope of the article, and include studies from higher and lower income countries. The author does a good job reviewing and comparing findings.

 

 The weaknesses are:

1. The economics of implementing more intensive and patient-centered approaches for end-of-life care are not discussed in depth, and if possible it would be interesting to explore this more, perhaps in a different paper. Economics do play a major role on how resources are allocated, and one wonders what are the specific implications in a low-resource setting.
2. The data was collected in a single institution, limiting generalizability.
3. There is a challenge when a single symptom attempts to represent the reason for a patient’s visit. Not uncommonly, some patients visit the ED for more than one complaint, and some symptoms clearly overlap.
4. A longitudinal follow-up was not performed in order to assess complaints that pose a higher risk of admission or poor outcome.

 

 In summary, this is a meaningful article that explores the particularities of an Academic Oncology Hospital in Brazil with high volume ED visits for emergencies. It is a simple, easily reproducible study. Its main strengths relies on the fact that the authors acquired primary data from patients as opposed to much larger studies from secondary data use, and it validates pain a the most important burden for cancer patients. The high rate of multiple visits stress the importance of this type of service and perhaps indicate room for improvement in the care of patients in their end of life with more intense palliative care. Hopefully, this work aids hospital from low-income settings to allocate the limited resources available in the health system. It would be interesting if the author could explore the combined symptoms at presentation but this may not be able to be recovered from when the data was collected.

I have read this submission. I believe that I have an appropriate level of expertise to confirm that it is of an acceptable scientific standard.

# Peer review: Referee response for version 1

**DOI:** 10.5256/f1000research.13677.r36629

**Published:** 2018-08-02

**Authors:** Evandro Dantas Bezerra

**Affiliations:** 1 Department of Medicine, University of Washington, Seattle, WA, USA

Very interesting topic and study design.

 Very well written.

 I approve this article without changes.

 I have the following minor comments:

- "Constitutional symptoms accounted for 17.3% of all visits; divided into **asthenia, fever, anorexia and malaise,** **responsible respectively for 46%, 40% and 12.5%** of the complaints in this category." **- **Not clear what was the prevalence of malaise, if you would like to report.
- Interesting that hematologic malignancies were only 5.8%. Is this cancer center focused in solid tumors? We know that hematologic cancers are less prevalent, but usually the patients are more ill.
- What was the prevalence of patients on chemotherapy? Recent surgery? Radiation?
- What was the rate of admission? If possible would be interesting to know if any specific complain or cancer were more likely to be admitted. I know this was not the focus of this paper, but this might interesting for second project.

I have read this submission. I believe that I have an appropriate level of expertise to confirm that it is of an acceptable scientific standard.
